# Exploring the intention to use e-cigarettes and its influencing factors among Thai non-formal education students: A cross-sectional study

**DOI:** 10.18332/tpc/211969

**Published:** 2026-01-28

**Authors:** Natchaya Palacheewa, Pramote Thangkratok

**Affiliations:** 1 Department of Community Health Nursing, Srisavarindhira Thai Red Cross Institute of Nursing, Bangkok, Thailand

**Keywords:** intention, e-cigarette, attitude, subjective norms, non-formal education students

## Abstract

**INTRODUCTION:**

Non-formal education students are a vulnerable group due to social influences and varying health literacy. However, evidence on their intention to use e-cigarettes and influencing factors is limited. This study aimed to investigate these intentions and associated factors among students in Bangkok, Thailand.

**METHODS:**

A cross-sectional survey was conducted among non-formal education students in Bangkok, Central Thailand, aged 13–24 years, who had never used e-cigarettes. A total of 116 participants were included in the study. Data were collected between 1 and 15 August 2025 using a self-administered structured questionnaire designed to assess knowledge, attitudes, subjective norms, perceived behavioral control, and intention to use e-cigarettes, based on the Theory of Planned Behavior. Descriptive statistics and multiple linear regression were applied to identify factors associated with the intention to use e-cigarettes.

**RESULTS:**

Only 2.6% of participants reported an intention to use e-cigarettes, 6.90% indicated that they might use them in the future, while 90.52% reported no intention to use e-cigarettes. Most participants demonstrated high knowledge of e-cigarettes (62.07%) and negative attitudes toward use (63.79%). Subjective norms were rated high (59.48%), and perceived behavioral control was very high (48.28%). Multiple linear regression analysis showed that attitudes (β=0.223; 95% CI: 0.03–0.41, p=0.025) and subjective norms (β=0.211; 95% CI: 0.02–0.39, p=0.032) had significant positive effects on the intention to use e-cigarettes. Overall, the model explained 15.0% of the variance (R^2^=0.150, adjusted R^2^=0.119; F=4.90, p=0.001).

**CONCLUSIONS:**

The findings highlight the importance of fostering negative attitudes toward e-cigarette use and reducing the influence of subjective norms among students. However, as this study employed a cross-sectional design, further longitudinal and interventional studies are needed to confirm these relationships and provide stronger evidence for the prevention of e-cigarette use among non-formal education students.

## INTRODUCTION

Electronic cigarettes (e-cigarettes, vaping, vape pens) are devices designed to deliver nicotine in the form of vapor through an electronic system^1^. They operate by heating a liquid, known as e-liquid, into an aerosol. This aerosol contains various chemicals, including propylene glycol, vegetable glycerin, nicotine, and flavoring agents^2^. Electronic cigarettes are rapidly increasing in popularity worldwide despite persistent uncertainty about their health impacts^3^. E-cigarettes were first invented in 2003 by a Chinese pharmacist as an alternative for individuals seeking to quit conventional smoking^4^. From their introduction, they were marketed as a healthier alternative to traditional cigarettes. In the United States, the number of e-cigarette users has steadily increased, rising from only 7 million in 2011 to 68 million in 2020, and reaching 82 million among the adult population in 2021^5^.

Global sales of e-cigarettes have also shown an upward trend, particularly among adolescents and young adults^6-8^. In the United States, the prevalence of e-cigarette use among adolescents and students increased by more than 78% between 2017 and 2018^9,10^. Similarly, a survey conducted in 27 European countries between 2012 and 2014 showed a significant rise in e-cigarette use, from 7% to 11.6%, accompanied by an increase in risk perception, from 27% to 51.6%^11^. In Thailand, the prevalence of e-cigarette use among adolescents (aged 13–15 years) increased from 3.3% in 2015 to 8.1% in 2021^12^. These findings highlight a significant upward trend in e-cigarette use among both adolescents and adults, at the global level as well as in Thailand, underscoring the need for further studies to address this growing public health concern in the future.

Globally, evidence shows a sharp increase in e-cigarette use, particularly among adolescents and young adults, yet available data remain limited and fragmented^13-16^. In Thailand, the prevalence of e-cigarette use among students (average age 13 years) was reported at 7.2% for ever use and 3.7% for current use^17^. However, most research has predominantly focused on students in formal schools and universities. Non-formal education students, by contrast, represent a unique and often overlooked group. They frequently face socioeconomic challenges, diverse peer influences, and limited access to structured health education, which makes them particularly vulnerable to adopting risky health behaviors such as e-cigarette use^18^.

Despite this vulnerability, little is known about their knowledge, attitudes, and behavioral intentions regarding e-cigarettes. The lack of empirical evidence indicates a gap in understanding the magnitude of e-cigarette use and the psychosocial factors influencing it among this population. Given the growing number of adolescents and young adults in non-formal education programs in Thailand, focusing on this group is essential^19^. Therefore, this study aimed to examine the factors influencing e-cigarette use among students enrolled in non-formal education programs in Thailand.

## METHODS

### Study design

This cross-sectional study was conducted between 1 and 15 August 2025 among non-formal education students enrolled in Bangkok Metropolitan Non-Formal and Informal Education Centers. The target population comprised students aged ≥13 years at the Khlong Sam Wa District Learning Encouragement Center, located in Bangkok, Central Thailand. The study site was selected through purposive sampling based on its geographical and demographic relevance. Khlong Sam Wa is situated within the Eastern Bangkok zone, an area experiencing significant socioeconomic development and digital exposure among adolescents. From a pool of five potential districts in this zone (Lat Krabang, Min Buri, Nong Chok, Prawet, and Khlong Sam Wa) one district was randomly selected using a simple random sampling method. Khlong Sam Wa District was selected as the study area, comprising one Non-Formal and Informal Education Center with a total student population of 722, including 24 in primary education, 338 in lower secondary education, and 360 in upper secondary education.

The sample size was calculated using G*Power software for multiple linear regression analysis, assuming a medium effect size (f^2^=0.15), statistical power of 0.80, and a significance level of 0.05, yielding a minimum required sample of 96 participants. To account for possible incomplete responses, 20% was added, resulting in a final sample size of 116 participants. The sample was proportionally stratified according to education level and gender distribution, comprising 4 primary students, 54 lower secondary students, and 58 upper secondary students. Inclusion criteria included enrollment in the center during academic years 2023–2025, aged ≥13 years, the ability to communicate effectively in Thai, no prior use of e-cigarettes, conventional cigarettes, or electronic hookah, and no existing respiratory conditions such as asthma or chronic cough. Exclusion criteria included incomplete responses, current treatment for tobacco-related substance use, or voluntary withdrawal at any point during the study.

### Data collection

Prior to data collection, formal permission was obtained from the center director. The researcher coordinated with homeroom teachers to identify eligible participants and facilitate logistics. Teachers were briefed on research objectives, ethical considerations, and their supporting roles in the data collection process. In cases where teachers declined participation, other willing staff were approached. If no suitable staff were available, the researcher made direct contact with students for informed consent. Data were collected in a designated, quiet meeting room after academic hours, ensuring participants could respond independently and confidentially. The researcher clearly explained the study objectives and procedures using age-appropriate language. For participants under 20 years of age, parental consent was required in accordance with Thai Civil and Commercial Code Section 19. Consent forms and questionnaires were distributed for students to take home, and signed documents were collected on a subsequent school day.

### Questionnaire

The research instrument was divided into six sections. The instruments were chosen based on: 1) their theoretical alignment with the Theory of Planned Behavior (TPB); 2) their previous validation in similar adolescent behavioral studies; and 3) their established reliability and cultural relevance to the Thai context.

The first section, the demographic questionnaire, collected information on age, sex, and education level, which were defined as categorical variables and treated as potential confounding factors in the analysis.

The second section measured knowledge regarding e-cigarette use. This scale was adapted from the Knowledge Regarding E-Cigarettes questionnaire developed by Alduraywish et al.^20^ and modified to fit the Thai context. It comprised 10 multiple-choice items with three response options: correct, incorrect, and ‘do not know’. Responses were scored as 1 point for a correct answer and 0 for an incorrect or ‘do not know’, producing a total score ranging from 0 to 10. Knowledge levels were classified based on the taxonomy of Bloom^21^ into: high (80–100%), moderate (60–79%), and low (<60%). The instrument development process was rigorous: 1) permission was obtained from the original author to translate the tool into Thai; 2) translation from English was performed to ensure conceptual equivalence; 3) the translated version was reviewed by three experts in nursing, health management technology, and bilingual proficiency to confirm content validity and cultural appropriateness; 4) all items achieved an index of item-objective congruence (IOC) >0.67; 5) back-translation into English was independently conducted by two bilingual experts; and 6) the second English version was verified by the original author. The finalized Thai version was pilot-tested with 20 students from Non-Formal Education Center, and psychometric testing demonstrated acceptable internal consistency, with a Kuder-Richardson 20 (KR-20) coefficient of 0.80. An example item from this section is: ‘E-cigarettes contain fewer harmful substances than traditional cigarettes’ (Correct answer: Incorrect).

The third section assessed attitudes toward e-cigarette use. This scale was adapted from the Electronic Cigarette Attitudes Survey developed by Alduraywish et al.^20^ and consisted of 10 items, including eight positive and two negative statements. Responses were rated on a 5-point Likert scale (1 = ‘strongly disagree’ to 5 = ‘strongly agree’), producing a total score between 10 and 50. The scores for the two negatively worded items in the attitude scale were reversed before analysis. Attitudes were categorized according to the criteria of Bloom^21^: positive (80–100%), neutral (60–79%), and negative (<60%). The instrument demonstrated strong psychometric properties, with all items achieving an IOC >0.67 and internal consistency reliability indicated by a Cronbach’s alpha of 0.93. An example item from this section is: ‘Using e-cigarettes can make people look more modern’ (Correct interpretation: Reflects a positive attitude toward e-cigarette use).

The fourth section evaluated subjective norms toward e-cigarette use. This scale was developed based on Theory of Planned Behavior^22^ and a review of relevant literature. It comprised six items rated on a 5-point Likert scale (1 = ‘strongly disagree’ to 5 = ‘strongly agree’), yielding a total score from 6 to 30. Mean scores were interpreted using class interval criteria^23^ categorized as: very low (1.00–1.80), low (1.81–2.60), moderate (2.61–3.40), high (3.41–4.20), and very high (4.21–5.00). The instrument demonstrated strong psychometric properties, with all items achieving an IOC >0.67 and internal consistency reliability indicated by a Cronbach’s alpha of 0.93. An example item from this section is: ‘People who are important to me think that I should not use e-cigarettes’ (Correct interpretation: Reflects perceived social pressure discouraging e-cigarette use).

The fifth section measured perceived behavioral control toward e-cigarette use, also based on the Theory of Planned Behavior^22^ and related literature. The scale included 10 items rated on a 5-point Likert scale, with possible scores ranging from 10 to 50. Mean scores were interpreted using the same interval criteria^23^. The instrument demonstrated strong psychometric properties, with all items achieving an IOC >0.67 and internal consistency reliability indicated by a Cronbach’s alpha of 0.89. An example item from this section is: ‘I am confident that I can refuse if someone offers me an e-cigarette’ (Correct interpretation: Reflects high perceived behavioral control over resisting e-cigarette use).

The sixth section assessed participants’ intention to use e-cigarettes. This scale was adapted from Cabral^24^ and consisted of five items with three response options: ‘yes’ (3 points), ‘maybe’ (2 points), and ‘no’ (1 point). Higher scores indicated a stronger intention to use e-cigarettes. For the purpose of analyzing the prevalence in this study, only the first question regarding intention to use e-cigarettes was used. The development process involved obtaining permission from the original author, translation into Thai with attention to conceptual equivalence, expert review for accuracy and cultural appropriateness, and evaluation of item-objective congruence (IOC), with all items scoring above 0.67. The instrument was back-translated by two bilingual experts and verified by the original author. The Thai version was pilot-tested with 20 non-formal education students. Psychometric testing confirmed strong quality, with a high internal consistency reliability, as demonstrated by a Cronbach’s alpha of 0.92.

An example item from this section is: ‘Do you intend to use e-cigarettes in the next six months?’ (Correct interpretation: Higher agreement reflects a stronger behavioral intention to use e-cigarettes).

### Statistical analysis

Data were coded and analyzed using SPSS version 29. Descriptive statistics of frequencies and percentages, and means and standard deviations were used to describe the demographic variables and study constructs. Inferential statistics, multivariable linear regression was used to examine the associations and predictive relationships between the independent variables (knowledge, attitudes, subjective norms, and perceived behavioral control) and the dependent variable (intention to use e-cigarettes). Prior to regression analysis, preliminary statistical tests, including independent t-tests and chi-squared tests, were performed to explore group differences. Statistical significance was set at p<0.05.

## RESULTS

The study included 116 respondents aged 13–32 years, with a mean age of 18.47 years (SD=3.81), indicating that half of the participants were aged ≥17 years. Regarding gender distribution, 63 participants (54.3%) were female, while 53 participants (45.7%) were male (Table 1).

**Table 1:** Demographic characteristics of participants of a cross-sectional study on intention to use e-cigarettes among non-formal education students, Khlong Sam Wa District, Bangkok, Thailand, August 2025 (N=116)

*Characteristics*	*Categories*	*n*	*%*
**Age** (years)	13–17	58	50.00
	18–24	46	39.66
	25–32	12	10.34
	Mean ± SD	18.47 ± 3.81	
**Gender**	Male	53	45.69
	Female	63	54.31
**Education level**	Primary school	24	20.69
	Lower secondary school	38	32.76
	Upper secondary school	54	46.55

Regarding knowledge about e-cigarettes, the majority of respondents demonstrated a high level of knowledge (n=72; 62.07%), followed by a moderate level (26.72%, n=31) and a low level (11.21%, n=13). In terms of attitudes toward e-cigarette use, most participants held negative attitudes (63.79%, n=74), while 31 respondents (26.72%) reported neutral attitudes, and only 11 respondents (9.48%) expressed positive attitudes. With respect to the influence of subjective norms, the largest proportion reported a high level of conformity (59.48%, n=69), followed by very high conformity (18.10%, n=21), moderate conformity (14.66%, n=17), low conformity (4.31%, n=5), and very low conformity (3.45%, n=4). Finally, perceived behavioral control was reported as very high by 56 respondents (48.28%), high by 32 respondents (27.59%), moderate by 16 respondents (13.79%), low by 5 respondents (4.31%), and very low by 7 respondents (6.03%). Among the participants, 2.59% reported an intention to use e-cigarettes, 6.90% indicated they might use them in the future, while 90.52% reported no intention to use e-cigarettes (Table 2).

**Table 2:** Levels of knowledge, attitudes, subjective norms, and perceived behavioral control regarding e-cigarettes, a cross-sectional study on intention to use e-cigarettes among non-formal education students, Khlong Sam Wa District, Bangkok, Thailand, August 2025 (N=116)

*Item*	*Categories*	*n*	*%*
**Level of knowledge about e-cigarettes**	High	72	62.07
	Moderate	31	26.72
	Low	13	11.21
**Level of attitudes toward e-cigarette use**	Negative	74	63.79
	Neutral	31	26.72
	Positive	11	9.48
**Level of subjective norms**	Very high	21	18.10
	High	69	59.48
	Moderate	17	14.66
	Low	5	4.31
	Very low	4	3.45
**Level of perceived behavioral control**	Very high	56	48.28
	High	32	27.59
	Moderate	16	13.79
	Low	5	4.31
	Very low	7	6.03
**Intention to use e-cigarettes**	Yes	3	2.59
	Maybe	8	6.90
	No	105	90.52

Multiple linear regression analysis showed that attitudes (β=0.223; 95% CI: 0.03–0.41, p=0.025) and subjective norms (β=0.211; 95% CI: 0.02–0.39, p=0.032) had significant positive effects on the intention to use e-cigarettes. In this model, the standardized beta coefficient (β) represents the strength and direction of the relationship between each independent variable and the dependent variable (intention). A positive β value indicates that as the predictor increases, the intention to use e-cigarettes also increases. Overall, the model explained 15.0% of the variance in intention (R^2^=0.150, adjusted R^2^=0.119; F=4.90, p=0.001) (Table 3).

**Table 3:** Multivariable linear regression analysis of knowledge, attitudes, subjective norms, and perceived behavioral control, a cross-sectional study on intention to use e-cigarettes among non-formal education students, Khlong Sam Wa District, Bangkok, Thailand, August 2025 (N=116)

*Predictor*	*β*	*b*	*t*	*p*	*95% CI*
Knowledge	-0.008	-0.01	-0.082	0.935	-0.21–0.19
Attitudes	0.059	0.223	2.27	0.025	0.03–0.41
Subjective norms	0.078	0.211	2.18	0.032	0.02–0.39
Perceived behavioral control	-0.019	-0.132	-1.16	0.249	-0.51–0.14

R²=0.150, adjusted R²=0.119; F=4.90, p=0.001.

## DISCUSSION

These results suggest that the overall intention to engage in e-cigarette use among students in non-formal education settings remains relatively low. This may reflect the effectiveness of health education campaigns, cultural norms, or community-based preventive efforts that discourage tobacco and e-cigarette use. In addition, Thailand has banned the import of electronic cigarettes (e-cigarettes) into the kingdom, and strict regulations prohibit their sale, advertisement, and marketing. The country enforces these measures through four main legal provisions: 1) prohibition of imports into the kingdom; 2) prohibition of sales through websites or online platforms; 3) prohibition of e-cigarette use in designated non-smoking areas; and 4) prohibition of possession, which is considered a legal offence. Such comprehensive restrictions are likely to reinforce preventive measures, limit accessibility, and reduce the attractiveness of e-cigarettes among young people^25^.

When compared with international studies, the prevalence of intention to use e-cigarettes in this sample appears lower. For example, previous studies reported higher proportions of adolescents and young adults expressing both current use and intention to use e-cigarettes^26,27^. Such differences may be influenced by variations in accessibility, marketing exposure, peer influence, and social acceptance of e-cigarettes across contexts. In Thailand, studies conducted among youths in the formal education system in the Northern Region have shown patterns similar to our findings. The intention to use e-cigarettes was found to be at a relatively low level, with cultural norms and strong anti-smoking policies potentially serving as protective factors^28^. The relatively high percentage of students reporting no intention to use e-cigarettes highlights an opportunity to strengthen preventive strategies by reinforcing positive attitudes and perceived behavioral control. According to the Theory of Planned Behavior (TPB)^22^, intention is shaped by knowledge, attitudes, subjective norms, and perceived behavioral control.

In this study, most students demonstrated moderate to high knowledge about e-cigarettes and negative attitudes toward their use, which may have contributed to the low levels of intention. Additionally, strong perceived control over behavior may serve as a protective factor against peer pressure and environmental influences^20^. However, the 6.90% of students who indicated uncertainty regarding future use remain a group of concern. This subgroup may be particularly vulnerable to external influences such as peer pressure, targeted marketing, or curiosity, which could increase the likelihood of future initiation. Tailored interventions focusing on strengthening decision-making skills, refusal strategies, and digital media literacy could be valuable in reducing this risk. Overall, the findings support the importance of early preventive measures and educational interventions in shaping knowledge, attitudes, and behavioral intentions. Public health programs should continue to emphasize the risks associated with e-cigarette use, while also monitoring emerging trends among youth populations to adapt interventions and policies accordingly.

This study investigated factors influencing the intention to use e-cigarettes among students in non-formal education centers in Bangkok, using the TPB. The results revealed that attitudes and subjective norms were significant predictors of the intention to use e-cigarettes, whereas knowledge and perceived behavioral control were not statistically significant. This suggests that students’ perceptions, beliefs, and social influences play a far greater role in shaping their behavioral intentions than factual awareness or perceived ability to resist the behavior. Specifically, students who hold more favorable attitudes toward e-cigarette use believing it to be modern, socially acceptable, or less harmful than traditional cigarettes tend to exhibit stronger intentions to use them. Likewise, when individuals perceive that significant others (such as friends, peers, or family members) approve of or engage in e-cigarette use, this social endorsement reinforces their intention to adopt similar behaviors. These findings emphasize that within the non-formal education context, where peer relationships and social belonging are highly influential, subjective norms can act as a powerful driver of behavioral decision-making.

In the Thai cultural context, where collectivism and social conformity play a central role, these results suggest that adolescents’ decisions are strongly shaped by their desire to maintain social acceptance and group identity. Therefore, interventions aimed at preventing e-cigarette use among non-formal education students should focus not only on improving knowledge but also on modifying attitudes and reducing social acceptability of e-cigarette use. Health education campaigns that highlight peer-led narratives, social responsibility, and the negative image of e-cigarettes may be more effective in influencing behavioral intentions than knowledge-based approaches alone. For instance, the study ‘Association between Personal Factors, Beliefs, and Attitudes towards E-cigarettes and Cigarettes Use among Thai Youths in the Central Region, Bangkok and its Perimeter’ reported that youths with positive beliefs and attitudes toward e-cigarettes were more likely to use both e-cigarettes and conventional cigarettes compared to those with negative beliefs and attitudes. Such evidence confirms that attitudinal and normative influences outweigh factual knowledge in predicting behavior^20^.

This finding supports previous evidence from China, which showed that young adult males with higher education possessed better knowledge of smoking hazards; however, this knowledge did not necessarily translate into healthier behavioral outcomes such as refraining from smoking. Overall, those findings indicated no statistically significant correlation between education level and the intention to quit smoking among young adult male smokers in China^29^. From a theoretical perspective, the present results reinforce the predictive validity of the TPB in explaining youth smoking behaviors. Attitudes and subjective norms accounted for 15% of the variance in intention, consistent with other TPB-based studies. The lack of significance for knowledge underscores the need for interventions that go beyond information provision. Programs that integrate digital media literacy, strengthen refusal skills, and engage peer leaders may be more effective than conventional health education approaches^30,31^.

### Limitations

This study has some limitations. First, the cross-sectional design restricts causal inference between determinants and intention. Second, reliance on self-reported questionnaires may introduce social desirability bias, particularly in sensitive behaviors such as smoking. Third, the relatively small sample size and focus on one district in Bangkok limit generalizability to other populations of non-formal education students. Future studies should adopt longitudinal or mixed-methods designs and include diverse settings to strengthen external validity.

## CONCLUSIONS

This study demonstrated that attitudes and subjective norms significantly influenced the intention to use e-cigarettes among non-formal education students in Bangkok, while knowledge and perceived behavioral control showed limited effects. These findings highlight the central role of social influence and personal beliefs in shaping smoking behaviors. Interventions should therefore focus on modifying attitudes, addressing misconceptions, and strengthening peer-based prevention strategies rather than relying solely on knowledge dissemination.

## Data Availability

The data supporting this research are available from the authors on reasonable request.
